# Exploring and Monitoring the Reasons for Hesitation with COVID-19 Vaccine Based on Social-Platform Text and Classification Algorithms

**DOI:** 10.3390/healthcare9101353

**Published:** 2021-10-12

**Authors:** Jingfang Liu, Shuangjinhua Lu, Caiying Lu

**Affiliations:** School of Management, Shanghai University, Shanghai 200444, China; jingfangliu@shu.edu.cn (J.L.); lucaiying2020@shu.edu.cn (C.L.)

**Keywords:** COVID-19 vaccine, vaccine hesitant, text classification

## Abstract

(1) Background: The COVID-19 pandemic is globally rampant, and it is the common goal of all countries to eliminate hesitation in taking the COVID-19 vaccine and achieve herd immunity as soon as possible. However, people are generally more hesitant about the COVID-19 vaccine than about other conventional vaccines, and exploring the specific reasons for hesitation with the COVID-19 vaccine is crucial. (2) Methods: this paper selected text data from a social platform to conduct qualitative analysis of the text to structure COVID-19 vaccine hesitancy reasons, and then conducted semiautomatic quantitative content analysis of the text through a supervised machine-learning method to classify them. (3) Results: on the basis of a large number of studies and news reports on vaccine hesitancy, we structured 12 types of the COVID-19 vaccine hesitancy reasons. Then, in the experiment, we conducted comparative analysis of three classifiers: support vector machine (SVM), logistic regression (LR), and naive Bayes classifier (NBC). Results show that the SVM classification model with TF-IDF and SMOTE had the best performance. (4) Conclusions: our study structured 12 types of COVID-19 vaccine hesitancy reasons through qualitative analysis, filling in the gaps of previous studies. At the same time, this work provides public health institutions with a monitoring tool to support efforts to mitigate and eliminate COVID-19 vaccine hesitancy.

## 1. Introduction

### 1.1. Background

Vaccine hesitation refers to people’s unsupportive or ambiguous attitude toward vaccines, including the outright denial of vaccines, the delay of vaccines, the acceptance of vaccines with misgivings, or partial vaccination [1]. In 2019, the World Health Organization listed “vaccine hesitation” as one of the top 10 threats to global health. It is globally widespread, especially in Western societies. At the beginning of 2020, the COVID-19 pandemic broke out globally, threatening the safety and health of all humankind.

Vaccination is one of the most effective public health interventions in the world [2]. The World Health Organization (WHO) recommends that governments respond positively to hot spots of vaccine hesitation on the basis of social and behavioral insights [3]. The sooner that herd immunity is achieved, the fewer mutations of the novel coronavirus would occur. This is urgent for the whole world.

Therefore, it is crucial to eliminate people’s hesitation about the COVID-19 vaccine and establish a herd-immunity barrier to alleviate the severe pandemic situation. Exploring the specific reasons for hesitation with the COVID-19 vaccine is the premise for eliminating hesitation. At the same time, most countries have a much higher aversion to the COVID-19 vaccine than to conventional vaccines [4]. This shows that countries around the world face unprecedented challenges in promoting the COVID-19 vaccine.

### 1.2. Literature Review

Scholars performed many studies and obtained findings regarding the reasons for vaccine hesitation. In this paper, we divide the reasons for vaccine hesitation into social and individual levels.

At the societal level, Aygun and Tortop (2020) [5] asserted that religious beliefs are one of the factors that contribute to vaccine hesitation and impede vaccination coverage. In addition to vaccine information sources [6], factors include the convenience of vaccination [7,8], vaccine shortages [9], and other vaccine service factors. Sociocultural structure [10], media misinformation [11,12], the humor of vaccine information [13], and social and spatial aggregation of families [14] were also associated with vaccine hesitation in previous studies.

At the individual level, studies found that various demographic characteristics, such as race [15,16], age [17,18,19], sex [16,18,19], education level [20], and economic status [21], are reasons for vaccine hesitation. In addition, other factors include the cognition of disease [22,23], parental attitudes [8,24], side effects [23,25,26], moral intuition [27], the seasonality and number of childbirths [28,29], trust in health professionals [23,30], vaccination history [31,32], and fear of needles [33].

The reasons for vaccine hesitancy were extensively discussed in previous studies at the societal and individual levels. Especially during the COVID-19 pandemic, scholars around the world developed a strong interest in the COVID-19 vaccine hesitancy, and these research results enriched the theoretical basis of vaccine hesitancy. In the field of vaccine hesitancy reasons, most research methods are mainly concentrated in questionnaires and face-to-face and telephone interviews. However, the application of machine-learning methods in this field is very scarce. The study used supervised machine learning to automatically categorize vaccine-related tweets on Twitter as against, for, or neutral [34] to help public health agencies in monitoring attitudes about vaccines in real-time. However, this work was not for the COVID-19 vaccine, nor can it be more detailed to identify the reasons for hesitation.

### 1.3. Objectives and Methodology

Mass vaccination campaigns to promote COVID-19 vaccines are onerous and difficult. Exploring and monitoring the specific reasons for the current hesitancy is a prerequisite for increasing vaccination coverage. Social platforms are able to capture real-time changes in public opinion on COVID-19 vaccination, potentially providing a quick, low-cost, and easy alternative to traditional questionnaire and interview methods. Monitoring content on social platforms is time-consuming and inefficient if it is manually identified by public health agency staff [35].

The motivation and goal of our work is to identify specific reasons for people’s hesitation about COVID-19 vaccines on a social platform. This fine-grained, semiautomatic classification provides a real-time monitoring tool for public health agencies, and our research is novel and more practical.

We firstly conducted qualitative analysis of the text to structuring vaccine hesitancy reasons, and then conducted semiautomatic quantitative content analysis of the text to classify them through supervised machine-learning methods.

### 1.4. Results and Contributions

On the basis of a large number of studies and news reports on vaccine hesitancy, we structured 12 types of the COVID-19 vaccine hesitancy reasons. Then, in the experiment, we conducted comparative analysis of three classifiers: support vector machine, logistic regression, and naive Bayes. Results showed that the Support Vector Machine (SVM) classification model with Term Valence-Inverse Document Frequency (TF-IDF) and Synthetic Minority Oversampling Technique (SMOTE) had the best performance. Our research enables the content identification of COVID-19 vaccine hesitancy reasons, and provides public health institutions with a fast, low-cost, and labor-saving monitoring tool, and provides strong support for alleviating and eliminating the COVID-19 vaccine hesitancy.

## 2. Methods

### 2.1. Data Collection

The obtained data in this study were from a large online social platform. We obtained 8302 texts from 4 April to 24 May 2021. In these texts, people present their reasons for hesitation about the COVID-19 vaccine.

After text screening, 7309 valid data could be used in the experiment, and the screening criteria are shown in Table 1. In the reserved text, particularly where a single text contained multiple reasons for hesitation, we split the text to ensure that each text contained only one reason. Lastly, the number of datasets was 7379.

### 2.2. Structuring COVID-19 Vaccine Hesitancy Reasons

On the basis of the international vaccine hesitancy literature and current news reports, we conducted qualitative analysis of relevant texts on a social platform, to construct 12 types of reasons for vaccine hesitancy. These reasons are the basis of subsequent classification models. Twelve types of vaccine hesitancy reasons are shown in Table 2 below.

Type 1: shortage of vaccines. In the previous literature [9], some scholars believe that, in view of the infectiousness of infectious diseases, a shortage of vaccines affects the health of individuals and populations through group effects. A more serious situation is that the shortage of vaccines could intensify the controversy of vaccination and become an argument for the antivaccine crowd.

Type 2: individual rights. Previous studies seldom discuss the relationship between personal rights and vaccine hesitation, and have mainly focused on the legal level. Yildirim argued that, even if mandatory vaccination is in the public interest, its legality principle should not be ignored [36]. Since herd immunity requires most citizens to complete vaccination against COVID-19 as soon as possible, on social platforms, some groups that were vaccinated or are willing to be vaccinated morally blackmail those who are hesitant about the vaccine, saying that those people are egoists. However, some people hesitating about vaccines refute this statement. They believe that it is a personal right not to vaccinate, and they have a strong aversion to such persecution and coercive speech and behavior, which hinders the process of eliminating vaccine hesitation.

Type 3: inconvenient inoculation. The linkage of community health services affects the scale and efficiency of immunization, such as living in remote areas, medical personnel shortages, and the indirect costs of immunization [37]. Inconvenient inoculation services [38] are also a reason for vaccine hesitation. Many people are unable to receive the COVID-19 vaccine on time because of the mismatch between the time that they leave work and the vaccination time, the difficulty of taking leave, long distances from the vaccination community, and the high indirect costs of vaccination (missed work and transportation costs).

Type 4: dissatisfaction with vaccine promotion. Propaganda strategies impact vaccination coverage. During the pandemic, people’s discussions of vaccine propaganda were of two types: (1) Vaccine spokesperson. An awareness campaign initiated by trusted scientific experts helps to increase the coverage of the COVID-19 vaccine [39]. On the other hand, studies showed that vaccine hesitancy is responsible for the widespread breakdown of trust between certain groups of people and elites and experts [40]. Our text data are more supportive of the latter, with many comments revealing distrust for Chinese vaccine experts or spokespersons. (2) Slogan or tagline. Studies showed that humorous messages reduced resistance to the measles vaccine, and thus resulted in less hesitation about the vaccine compared to serious messages [13]. On social platforms, people prefer humorous and easy-to-understand propaganda messages. However, some people think that these messages are too informal and make them feel embarrassed.

Type 5: vaccine effect and validity period. New studies showed that the effect of the COVID-19 vaccine on obese people may be poor, which may make obese people hesitant to be vaccinated [41]. An increase in vaccine efficacy from 50% to 90% was associated with a 10% increase in the marginal mean willingness to vaccinate (from 0.51 to 0.61) [42]. However, due to the lack of clinical data, the validity of the COVID-19 vaccine has not been determined. As a result, many people worry about the short validity period, which is an important reason for hesitation.

Type 6: living in low-risk areas. Risk perception refers to the subjective identification of risks that individuals may face, which is determined by uncertainty and fear [43]. A recent study showed that, geographical distance significantly impacts social risks perceived by local communities [44], and the greater the distance from high-risk areas is, the lower people’s perceived risks are. When a person is considering whether to be vaccinated, concerns about vaccine safety may outweigh perceptions of disease risk [45].

Type 7: antiviral mutation ability of the vaccines. It is encouraging that the latest study shows that the quality of immunity caused by the COVID-19 vaccine is superior to that caused by natural infection [47]. Therefore, people need not worry too much about the antiviral mutation ability of vaccines. The current COVID-19 vaccine is still an effective means to prevent the novel coronavirus. However, there are concerns that new coronavirus vaccine is ineffective against mutated viruses. The formation of risk perception of uncertain risks is related to information. When people face unknown or uncertain disasters, they tend to search for information [46], and the public can only rely on the media to collect information. People’s concerns about the antiviral mutation ability of the vaccines are probably due to the anxiety caused by a large number of false reports and discussions on social media.

Type 8: underlying diseases. According to media reports and field interviews, many people with underlying diseases worry about complications after being vaccinated. Underlying diseases in our text data include chronic diseases such as hypertension, eczema, diabetes, hyperthyroidism, cough, urticaria, heart disease, and bronchitis. However, in a study, chronic disease was associated with higher vaccine acceptability (non-COVID-19 vaccines) [48], which is contrary to our observation.

Type 9: adverse reactions and side effects. Severe adverse reactions or side effects from vaccination are the most common reason people refuse vaccination. Previous studies discussed the relationship between side effects and vaccine hesitation. For COVID-19 vaccines, studies showed that a reduction in the incidence of side effects increases the marginal mean willingness to receive the vaccine by 4% (from 0.54 to 0.58) [42].

Type 10: women during pregnancy and lactation. Women during pregnancy and lactation have more concerns about vaccination, especially for new vaccines that have recently entered the market such as the COVID-19 vaccine, and clinical data are still relatively scarce. However, in a Japanese study, experts collected global reports of adverse reactions to COVID-19 vaccination in pregnant women or women wishing to become pregnant and advocated guidelines such as not excluding pregnant women from vaccination [49], a policy recommendation that was adopted by the Japanese government. In some countries, this group was not included in the COVID-19 vaccination program.

Type 11: fear of needles. The fear of needles appears around age 5 and leads to the avoidance of healthcare [33], including vaccine hesitancy. Addressing the pain associated with vaccination is important to prevent needle phobia [50]. It is a challenge for medical staff to correctly and effectively use injection techniques to relieve the pain of vaccination, and thereby eliminate vaccine hesitation.

Type 12: allergy sufferers. Allergic reactions caused by the vaccine itself or vaccine components are rare, but this situation can be serious or even fatal when it occurs [51]. For the COVID-19 vaccine, there were two allergy-related contraindication conditions: (1) those who were allergic to the active ingredient of the vaccine, any inactive ingredient, or substances used in the production process, or those who were allergic to previous vaccination against similar vaccines; (2) people with previous severe allergic reactions to vaccines (such as acute allergic reactions, angioneurotic edema, and dyspnea). However, experts said that, in addition to the above allergic contraindications, allergies to pollen, willow catkin, and antibiotics (penicillin, cephalosporin) are not contraindications to the new coronavirus vaccine, and such people can be vaccinated against it. However, our observations on social-platform texts showed that there was still some hesitation toward the COVID-19 vaccine in these groups.

### 2.3. Text Preprocessing

**Figure 1:**
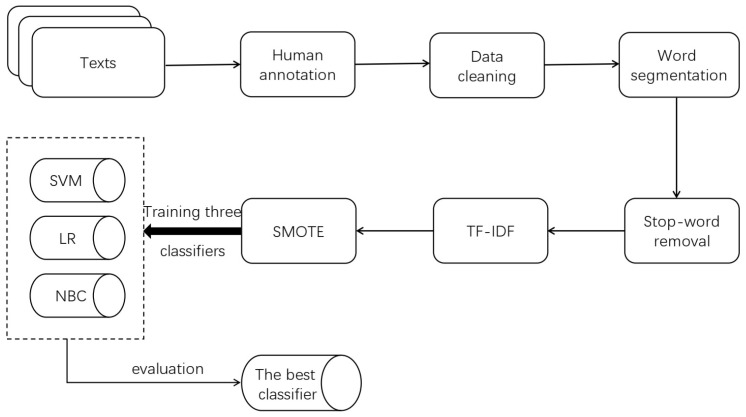
Experiment flow diagram.

The experimental process is shown in Figure 1. In natural-language processing, the combination of different preprocessing methods is very important to find the best classification rate. According to the experimental thought of Eler, Grosa, et al., (2018) [52], introducing TF-IDF and SMOTE is the best preprocessing combination in our experiment. The specific preprocessing steps were as follows.

Step 1: A team of 3 members was set up to manually annotate 7379 texts. We independently employed three graduate students who majored in information management and information systems. Before annotation, everyone was asked to read the entire text to familiarize themselves with the contents. Furthermore, training sessions were introduced to ensure that team members fully understood the meaning of each class. Each text was labeled by two members (the label type had to be one of the 12 reasons in Table 2). If the two labels of a text were the same, it was qualified. If not, the third person judged the final class.

Step 2: Data cleaning. We deleted all symbols except numbers, letters, and Chinese characters, and converted traditional Chinese characters in the text into simplified Chinese characters.

Step 3: Word segmentation, which is a necessary step in natural-language processing. Unlike English, Chinese sentences usually have no separation of words. Therefore, we used the Jieba library, a Chinese word segmentation tool, to process the text.

Step 4: Stop-word removal. After word segmentation, all words were extracted from the text, but some were redundant. These words were both ineffective for training the classifier, and increased the amount of computation. The elimination of stop words could help us optimize the results of word segmentation, thus improving the efficiency and effect of classification.

Step 5: The term valence-inverse document frequency (TF-IDF) model [53] was adopted to calculate the weight of each word. TF-IDF is a natural-language processing technology based on word frequency statistics that is widely used in text mining in news-media, social-science, medical, and other fields. It considers both the occurrence frequency and the rarity of words in the corpus. The higher the frequency and scarcity of a word are in the corpus, the greater the weight of the word.

**Figure 2:**
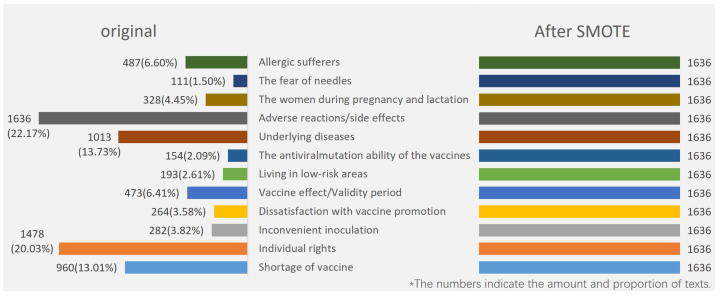
Original and oversampled sample distribution of 12 classes.

Step 6: The SMOTE algorithm was used to oversample some minority classes. As shown in Figure 2, the distribution of the original text quantity in the 12 categories was very uneven. The “adverse reactions/side effects” category had the largest number, with 1636 texts (22.17% of the total texts). The fewest were in “fear of needles”, which had 111 texts (only 1.50% of the total texts). Sample imbalance is very common and reasonable in the real world. For example, in the binary classification task of identifying people with cancer, the number of samples without cancer must be much larger than the number of samples with cancer. However, the problem of sample imbalance seriously affects the training effect of the classifier. The classifier overfits the majority class and underfits the minority class. To solve this problem, we needed to oversample a few classes.

**Figure 3:**
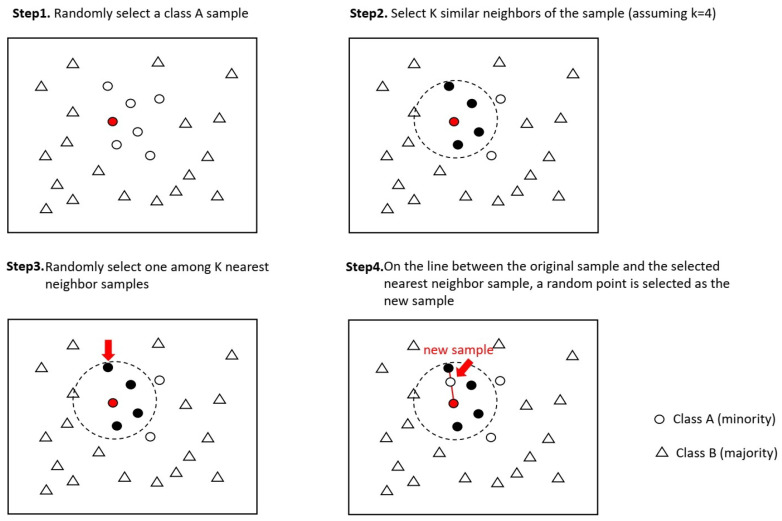
Concept of SMOTE algorithm.

SMOTE [54] is the oversampling algorithm put forward by Nitesh et al. that widely and effectively solves the sample imbalance problem in multiclassification tasks. The concept of the SMOTE algorithm is as shown in Figure 3. For each sample of the minority class, first find K neighbor samples of the same class (using Euclidean distance); then, randomly select one of the K neighbor samples. On the line between the original sample and the selected nearest-neighbor sample, a random point is selected as the new sample. The new sample has the same label as that of the original sample. Compared with the traditional random oversampling method of simply copying samples, SMOTE has better generalization ability. We set 1636 of the maximal class as the target number and used the SMOTE algorithm to increase all other minority classes to 1636, with a total of 19,632 texts in the dataset.

### 2.4. Training Classifier

In this section, 19,632 texts are randomly divided into training and test sets at a 4:1 ratio. The training and test sets are mutually exclusive. Each sample in the dataset has only one label. We trained three classical classifiers, support vector machine (SVM), logistic regression (LR), and naive Bayes classifier (NBC) through the training set samples, and then evaluated their performance on the test set to find the classifier with the best performance. We used the best classifier as a tool to monitor the reasons for hesitation with COVID-19 vaccine.

SVM [55] is a statistics-based machine-learning method developed by Vapnik, and it is widely used in natural-language processing, portrait recognition, handwriting recognition, and other fields. SVM has much superior performance on small-sample classification tasks. However, the standard SVM can only solve the binary classification problem. To achieve the multiclassification of hesitation reasons in this paper, we constructed multiple decision boundaries. The idea of multiclassification learning is to split multiclassification tasks into multiple binary classification tasks. There are three splitting strategies: one vs one, one vs all, and many vs many. The adopted strategy in this article was one vs all. In this paper, our category was 12, so we built 12 binary classifiers, and took samples of one class as positive samples, and samples of the remaining eleven classes as negative samples to train 12 classifiers. When predicting a certain sample of the test set, if only one classifier predicts positive samples, the sample belongs to this class; if more than one classifiers predict positive samples, the class with the highest confidence is selected as the final result. In the experiment, SVM parameters were set as follows: kernel = ‘Linear’, probability = True. In this paper, the linear function was selected as the kernel function of SVM, and the probability parameter was whether to use logistic regression to calibrate the SVM score after the completion of training. In addition, this paper used logistic regression and naive Bayes classifier for comparative study.

## 3. Results

As shown in Table 3, the performance of the three classifiers was evaluated through four indicators: the overall accuracy, precision, recall, and *F*1 *score*. The overall accuracy is the ratio of the number of correctly classified samples to the total number of samples. Precision is to calculate the ratio between the number of correctly classified samples in each class and the number of samples in each class, respectively, and then calculate the average precision. Recall refers to the proportion of positive samples that are actually positive among all predicted positive samples. In machine-learning tasks, the ideal situation is for both precision and recall to be high, but they are often a contradictory pair of metrics. As shown in the Equation (1), we also used *F*1 *score*, which is defined as a harmonic average based on precision (*P*) and recall (*R*), to evaluate the overall performance of the classifier.

(1) F1score=(2PR)/(P+R)

Experiments showed that SVM was superior to LR and NBC in all four indicators. F1 score was 86.8373%, and overall accuracy was 87.2422%. The performance of LR was slightly lower than that of SVM, while the performance of NBC was the worst, with all indices below 75%.

**Figure 4:**
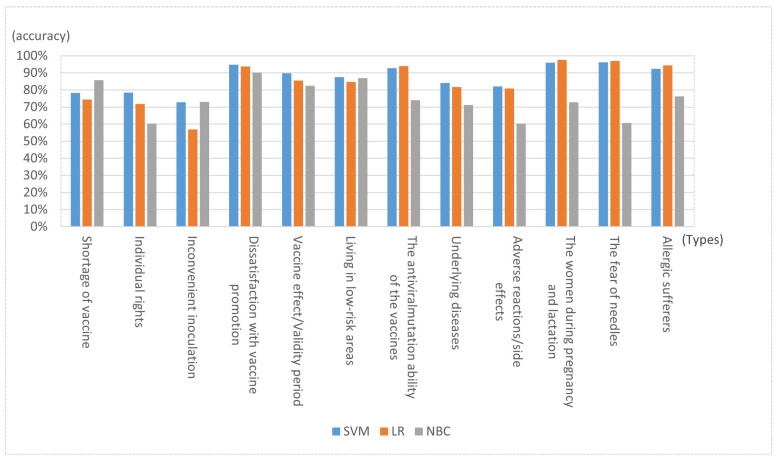
Accuracy of each class on three classifiers.

Figure 4 shows the accuracy of each class on the three classifiers. For the three classifiers, the performance of SVM and LR in 12 classes was relatively stable and good. The accuracy of SVM was better than that of LR in most categories, but the classification performance of NBC in 12 classes greatly fluctuated. NBC outperformed the two other classifiers in the “shortage of vaccine” and “inconvenient inoculation” classes. The accuracy of “dissatisfaction with vaccine promotion” was also over 90%, and “living in low-risk areas” was 87.037037%, which was close to the performance of SVM and LR. However, accuracy in other categories was poor.

Regarding the 12 classes, SVM with the best performance was taken as an example; the classification accuracy of the first three classes was not high because the class of “individual rights ”contained a large number of ironic sentences that were difficult for machine learning to deal with, thus reducing accuracy. The main reason for the low accuracy of “shortage of vaccine” and “ inconvenient inoculation ” is that the text contents of the two categories were similar and difficult to distinguish. The two classes with the highest accuracy were “women during pregnancy and lactation” and “fear of needles”. The content features of these two classes were very distinct, and keywords were well-distinguished from other classes. For example, the former text generally contains “pregnancy”, “breastfeeding”, and “pregnancy preparation”.

## 4. Conclusions

In this paper, qualitative analysis was used to construct 12 types of vaccine hesitancy reasons. We proposed an automatic classification method that introduces TF-IDF and SMOTE, which can realize the real-time monitoring of vaccine hesitancy reasons. Our research provides public health institutions with a fast, low-cost, and labor-saving monitoring tool, and provides strong support for alleviating and eliminating COVID-19 vaccine hesitancy. Three classical classifiers, namely, SVM, LR, and NBC, were evaluated in the classification stage. Our results showed that SVM performed well in all indicators.

## 5. Contributions and Future Work

### 5.1. Academic Contribution

In this study, we obtained a large number of discussion texts from a large social platform on the hesitation of COVID-19 vaccine, and constructed a dataset describing hesitation for the COVID-19 vaccine that provides a data basis for subsequent researchers.

This study summarized 12 types of reasons for hesitation to COVID-19 vaccines. Among them, there were reasons consistent with previous conventional vaccine hesitation studies, and there were also particular reasons for the COVID-19 pandemic. Our research results both supplement the research on reasons for vaccine hesitation and provide the main reasons why people hesitated about the COVID-19 vaccine during the pandemic. This is very meaningful for scholars to conduct future cross-border comparative research.

We propose a high-performance automatic classification method that can deal with the common sample imbalance problem in the vaccine hesitation field and can automatically identify the COVID-19 vaccine hesitancy reasons on social platforms. Our research framework applies to COVID-19 vaccine-hesitancy text analysis that extends to social platforms in different countries.

### 5.2. Practical Significance

On the basis of the relevant literature and news reports, this study explored 12 types of reasons for the COVID-19 vaccine hesitancy, which can provide directional reference and suggestions for public health institutions to formulate vaccine policies and immediately optimize vaccination services.

In this paper, we proposed an automatic classification method that introduces TF-IDF and SMOTE, which can realize the real-time monitoring of vaccine hesitancy reasons. For example, public health institutions can use our model to quickly identify the major reasons for hesitation on social platforms within a month, and this can help public health institutions in developing appropriate communication and information strategies (e.g., inviting vaccine spokespersons or authoritative doctors to conduct professional science popularization) to address public doubts and concerns about COVID-19 vaccines.

### 5.3. Future Work

The data source of this study is a large social platform. However, most of the participants on this website are teenagers and young people. The elderly and children are less involved. In future research, other data sources can be added or mixed-method research can be conducted in combination with interviews and other forms.

## Figures and Tables

**Table 1:** Screening criteria of texts.

The Content of Texts	Example Sentences	Keep/Delete
Mention one or more types of reasons for the COVID-19 vaccine hesitancy (among 12 types, shown in Table 2 below).	The main reason for my hesitation is that I really don’t know whether the vaccine is safe or not, and the validity period after vaccination is only half a year, so I have no courage. I am really afraid that the side effects will be too serious. One of my colleagues got vaccinated and had a fever every two days.	Keep
Only express hesitation without mentioning specific reasons.	The community has told us to get vaccinated, but I’m still on the fence.	Delete
Personal attacks or invective.	Those who do not want to be vaccinated should die!	Delete
Express that someone was vaccinated, without any hesitation.	I got vaccinated last week, and I’ll be there on time for the second dose.	Delete
Discuss the status of the epidemic or the origin of the virus.	The epidemic is currently under control, although there are occasional clusters of cases in some areas.	Delete

**Table 2:** Structuring COVID-19 vaccine hesitancy reasons.

Types	Names	Example Sentences	References
1	Shortage of vaccine	I received the first dose, and the second dose was always out of stock, so I was informed to reinoculate after the time.	literature [9]
2	Individual rights	Voluntary to mandatory? Vaccination or not depends on personal desire. Why morally blackmail?!	literature [36]
3	Inconvenient inoculatio	In small cities, the COVID-19 vaccines are available every working day, but not on weekends. Office workers have to take time off for the vaccine, and many of them were not vaccinated until now.	literature [37,38]
4	Dissatisfaction with vaccine promotion	(1) Is he a doctor or a social media influencer now? Shouldn’t scientists be serious about their research? (2) I thought the slogan was terrible and it didn’t convince me to get vaccinated.	literature [13,39,40]
5	Vaccine effect/Validity period	Is our vaccine safe and effective? This is the point that experts should pay attention to, right? Otherwise, when all people are vaccinated, some people will be injured.	literature [41,42]
6	Living in low-risk areas	After vaccination, it can only protect against the virus for a few months. I am in a low-risk area, so it’s truly unnecessary.	literature [43,44,45]
7	The antiviral-mutation ability of the vaccines	The current vaccine is developed against the previous virus. Is it effective against mutated viruses?	literature [46,47]
8	Underlying diseases	I truly want to get vaccinated, but I cannot. Eczema, chronic urticaria, diabetes...	literature [48]
9	Adverse reactions/side effects	My husband was vaccinated. He has a low fever for two days. He is lethargic and weak. It took him about a week to recover.	literature [42]
10	The women during pregnancy and lactation	I’m preparing for pregnancy, so I cannot get vaccinated, and then I cannot get vaccinated during breastfeeding. So I cannot get vaccinated within two years.	literature [49]
11	The fear of needles	In addition to I’m afraid of injections, nothing can stop my vaccination.	literature [33,50]
12	Allergic sufferers	I am a man of poor immunity, over the years I have allergic rhinitis, especially when the autumn, so I dare not vaccinated recently.	literature [51]

**Table 3:** Overall accuracy, precision, recall, F1 score of three classifiers.

Classifier Name	Overall Accuracy	Precision	Recall	F1-Score
SVM	0.872422	0.871475	0.870964	0.868373
LR	0.833206	0.844130	0.832090	0.833418
NBC	0.746117	0.745158	0.744181	0.727212

## Data Availability

Not applicable.
